# Intellectual and Physical Disability and Risk of COVID-19 Infection, Hospitalisation, and Mortality: A National Cohort of 3.7 Million Adults in Scotland

**DOI:** 10.1007/s44197-026-00581-4

**Published:** 2026-05-21

**Authors:** Yusuff Adebayo Adebisi, Nick Bailey, Angela Henderson, Chris Dibben, Serena Pattaro

**Affiliations:** 1 Administrative Data Research Scotland, School of Social and Political Sciences, University of Glasgow, Glasgow, UK; 2 School of Health and Wellbeing, University of Glasgow, Glasgow, UK; 3 Administrative Data Research Scotland, School of Geosciences, University of Edinburgh, Edinburgh, UK; 4 School of Social and Political Sciences, College of Social Sciences, University of Glasgow, 40 Bute Gardens, Glasgow, G12 8RT UK

**Keywords:** COVID-19, SARS-CoV-2, Disability, Intellectual disability, Physical disability, Infection, Hospitalisation, Mortality, Scotland

## Abstract

**Background:**

Disabled people experienced disproportionately poor outcomes during the COVID-19 pandemic, but evidence remains uneven across disability status and across stages of the disease pathway. Few population-wide studies have simultaneously examined infection, hospitalisation, and mortality while distinguishing intellectual disability from physical disability. The aim of this study was to quantify associations between disability status and COVID-19 outcomes in a national Scottish cohort.

**Methods:**

This population-wide retrospective cohort study included 3,719,651 adults aged ≥ 16 years alive and resident in Scotland on 1 March 2020. Disability status was derived from the 2011 Scottish Census and categorised as intellectual disability (*n* = 17,354), physical disability (*n* = 377,706), or no recorded intellectual or physical disability (comparison group; *n* = 3,324,591). Participants were followed from 1 March 2020 until 30 April 2022 for first laboratory-confirmed SARS-CoV-2 infection, first COVID-19 hospitalisation, and COVID-19-related mortality. Associations were estimated using Cox proportional hazards models with sequential adjustment for demographic factors, socioeconomic factors, health-related factors, and COVID-19 vaccination status.

**Results:**

During follow-up, 306,343 participants had a first recorded SARS-CoV-2 infection, 22,945 experienced a first COVID-19 hospitalisation, and 12,893 died from COVID-19-related causes. Crude incidence rates for first recorded SARS-CoV-2 infection were highest among adults with intellectual disability, whereas crude incidence rates for COVID-19 hospitalisation and mortality were highest among adults with physical disability. In fully adjusted models, compared with adults with no recorded intellectual or physical disability, intellectual disability was associated with higher hazards of infection (HR 2.65, 95% CI 2.57–2.73), hospitalisation (HR 1.60, 95% CI 1.40–1.83), and mortality (HR 1.58, 95% CI 1.30–1.91). Physical disability was also associated with higher hazards of infection (HR 1.60, 95% CI 1.58–1.62), hospitalisation (HR 1.16, 95% CI 1.12–1.19), and mortality (HR 1.23, 95% CI 1.19–1.28). Across all three outcomes, hazards were higher for intellectual than physical disability.

**Conclusions:**

Both intellectual and physical disability were independently associated with increased risk across the COVID-19 disease pathway, with stronger associations observed for intellectual disability. These findings support disability-inclusive pandemic preparedness and prevention strategies that recognise heterogeneity of risk within the disabled population and promote equitable access to timely care.

**Supplementary Information:**

The online version contains supplementary material available at 10.1007/s44197-026-00581-4.

## Introduction

The COVID-19 pandemic caused substantial morbidity and mortality globally and brought renewed attention to longstanding inequalities in exposure, vulnerability, and access to healthcare [1, 2]. From early in the pandemic, disabled people were identified as a population experiencing disproportionately poor outcomes, including higher risks of death [3, 4]. These disparities are plausibly shaped by multiple, intersecting factors, including a higher prevalence of chronic health conditions, potential intrinsic biological susceptibility (for example, immunological dysregulation or respiratory impairment in specific conditions), greater reliance on health and social care services, and structural barriers to timely and accessible healthcare [5, 6]. Barriers may include difficulties accessing information, lack of reasonable adjustments, and delays in clinical recognition or escalation of care [6, 7]. Patterns of regular support, including close contact with paid carers or support workers from outside the household, may further increase exposure risk in some groups [8]. At the same time, public health responses were not always designed with disabled populations in mind, potentially exacerbating existing inequalities [9]. Importantly, disability is not a single or uniform construct, and risks are unlikely to be evenly distributed across all disabled people. Understanding how COVID-19 risk varies across different disability types and across stages of the disease pathway is therefore essential for equitable public health planning.

Evidence relating to people with intellectual disability has been particularly consistent in demonstrating elevated risks of severe COVID-19 outcomes [10, 11]. Large population-based studies in England have reported markedly increased risks of COVID-19 hospital admission and mortality among disabled people compared with the general population [12]. These elevated risks persisted even after adjustment for age, sex, and other key demographic factors, indicating that excess risk could not be fully explained by population structure alone. Scotland-based studies have similarly identified higher risks of infection and adverse COVID-19 outcomes among adults with intellectual disabilities [13]. Across settings, these findings suggest that vulnerability among people with intellectual disability may extend beyond mortality to include increased susceptibility to infection and progression to severe disease once infected. Proposed explanations include higher prevalence of multimorbidity, communication barriers that may delay care-seeking or diagnosis, and increased exposure through supported living or residential care environments [14, 15]. Like physical disability, intellectual disability is also socially patterned: prevalence is higher in more deprived areas and varies by ethnicity, with these gradients evident from childhood [16]. These social patterns shape exposure to adverse environmental conditions, access to services, and underlying health, and are themselves linked to differential COVID-19 risk. However, reported effect sizes vary across studies, reflecting differences in population definitions, exposure measurement, outcome ascertainment, and analytical strategies [11]. This heterogeneity complicates direct comparison across settings and limits the ability to draw clear, locally relevant conclusions.

For physical disability, the evidence base is broader but often more difficult to interpret. Physical disability is strongly patterned by age, socioeconomic deprivation, and underlying health status [17], all of which are themselves powerful determinants of COVID-19 outcomes. UK analyses using linked population data have reported higher COVID-19 mortality among disabled people [12, 13], but many studies treat disability as a single exposure or do not clearly distinguish physical disability from intellectual or other forms of disability [11]. As a result, it remains unclear whether observed associations reflect risks specific to physical disability or broader patterns driven by ageing and comorbidity. In addition, many studies focus primarily on mortality outcomes [11], which cannot determine whether disparities arise from higher infection risk, greater severity following infection, or both. A disease-pathway perspective that considers infection, hospitalisation, and mortality together provides a more informative framework for identifying where inequalities emerge. Such an approach can clarify whether prevention, early detection, or clinical management should be prioritised for different groups. Despite the availability of high-quality national COVID-19 surveillance data, Scotland-specific evidence that contrasts intellectual and physical disability within the same population and across multiple COVID-19 outcomes remains comparatively limited [11].

The aim of this study was to examine how intellectual disability and physical disability are associated with the risk of COVID-19 outcomes among adults in Scotland. Specifically, we aimed to estimate differences in the risk of first laboratory-confirmed COVID-19 infection, first COVID-19 hospitalisation, and COVID-19-related mortality between adults with intellectual disability, adults with physical disability, and a comparison group with neither intellectual nor physical disability. By providing Scotland-specific estimates across the COVID-19 disease pathway, this study aims to strengthen the evidence base on disability-related inequalities.

## Methods

### Study Design, Data Sources, and Study Population

**Fig. 1:**
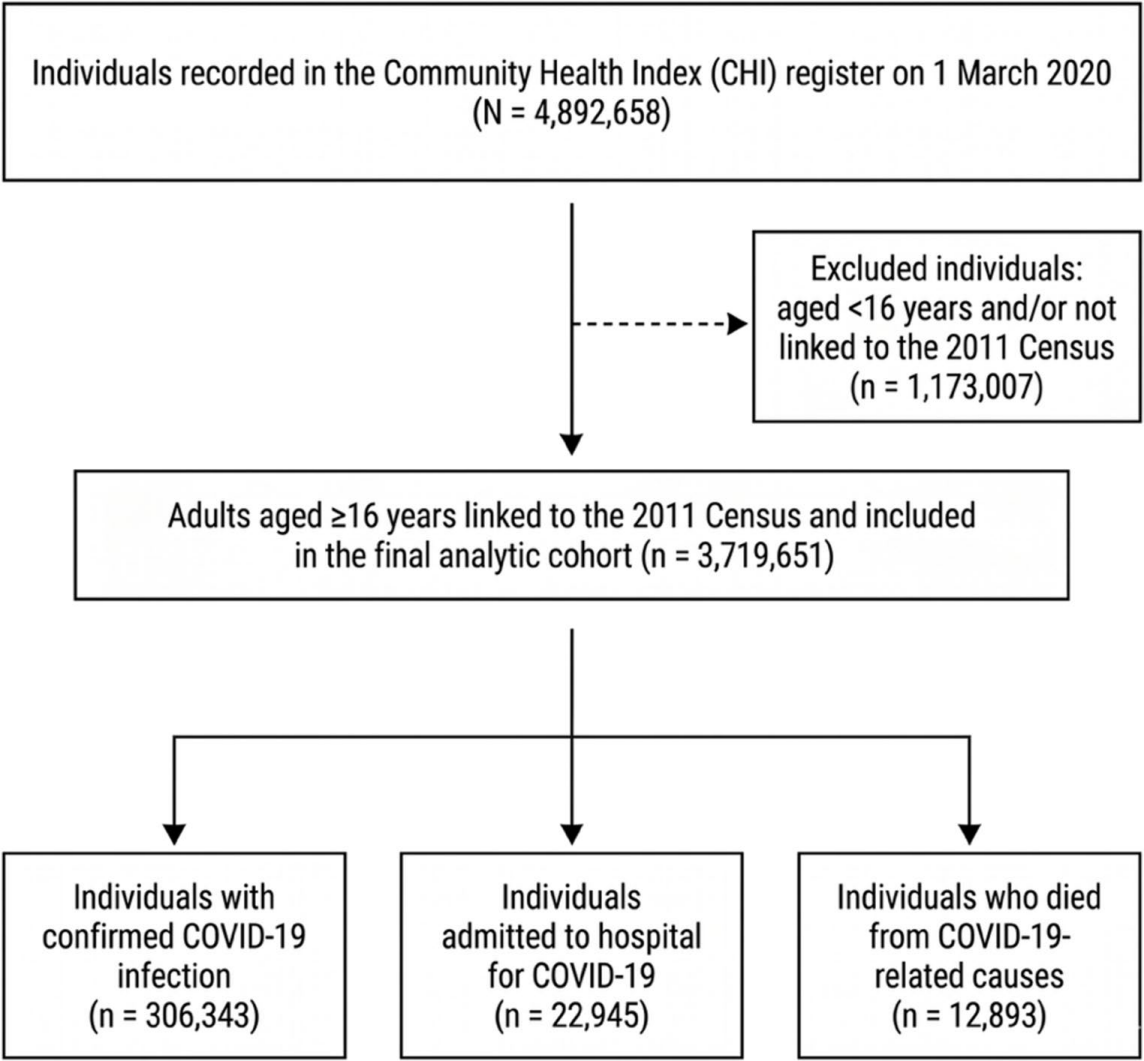
Sample Derivation

We conducted a population-wide retrospective cohort study using linked national administrative data in Scotland. Data linkage and analysis were undertaken within Scotland’s national secure data environment in accordance with established information governance and data protection requirements [18]. The study population comprised adults aged 16 years or older who were alive and resident in Scotland on 1 March 2020 (the cohort index date; baseline). Individuals were identified from the Community Health Index (CHI) register and linked to the 2011 Scottish Census to obtain baseline sociodemographic characteristics and self-reported disability status (Fig. 1).

Of 4,892,658 individuals recorded in CHI at the index date, those aged under 16 years were excluded. Among age-eligible adults, 3,719,651 individuals (90.0%) were successfully linked to Census records and constituted the final analytic cohort. Because the CHI register covers all Scottish residents irrespective of where they live, the cohort was population-wide and included adults across all residence types, including those in private households and in communal establishments such as care homes and supported living settings. The remaining approximately 10% of age-eligible CHI records that could not be linked to the 2011 Census most likely reflect linkage limitations, such as incomplete or non-standard address information, residential mobility, and changes in administrative records, rather than ordinary item-level missingness; imputation was not pursued because disability status and several baseline covariates are themselves derived from the Census.

### Exposure

The primary exposure was disability status, derived from self-reported long-term conditions recorded in the 2011 Scottish Census [19]. Individuals were classified into three mutually exclusive groups using a sequential approach:

1. Intellectual disability, identified by Census responses indicating learning disability, which in the UK context denotes a condition arising in childhood that significantly affects intellectual and adaptive functioning. Individuals in this category may also report co-occurring physical or sensory impairments (i.e. physical disability as defined in the next group).
2. Physical disability, defined as reported physical and/or sensory impairment (including deafness or partial hearing loss, and blindness or partial sight loss) among individuals who did not report intellectual disability. The term “physical disability” is used throughout this paper as shorthand for this combined category of physical and/or sensory impairment, in line with the protocol paper for this study.
3. Comparison group, comprising individuals without reported intellectual or physical disability. This group may include individuals reporting other long-term conditions not used to define the primary disability exposure.

Disability status was treated as a fixed baseline exposure. Within the cohort of 3,719,651 adults, 17,354 individuals (0.5%) were classified as having intellectual disability, 377,706 (10.2%) as having physical disability, and 3,324,591 (89.4%) as the comparison group.

### Outcomes

Three COVID-19 outcomes were examined: laboratory-confirmed infection, hospitalisation, and mortality. Outcomes were ascertained longitudinally between 1 March 2020 and 30 April 2022. Further operational details on outcome derivation, data sources, and coding rules are provided in Supplementary Table S1.

COVID-19 infection was defined as the first recorded laboratory-confirmed positive SARS-CoV-2 test during follow-up. Positive test results were identified using COVID-19 testing datasets integrated within the Public Health Scotland COVID-19 Research Database. COVID-19 hospitalisation was defined as the first qualifying inpatient admission recorded in the Scottish Morbidity Record 01 (SMR01). Hospitalisations were identified as admissions with COVID-19 recorded as a primary or secondary diagnosis, or admissions occurring within 14 days of a laboratory-confirmed positive SARS-CoV-2 test. COVID-19 mortality was ascertained using national death registration records from the National Records of Scotland (Supplementary **Table S1**). Deaths were classified as COVID-19–related if COVID-19 was recorded as an underlying or contributory cause of death, or if death occurred within 28 days of a laboratory-confirmed SARS-CoV-2 infection.

### Covariates

Covariates were specified a priori based on established associations with disability and COVID-19 outcomes and were defined at baseline (cohort index date: 1 March 2020), unless otherwise stated (Supplementary Figure S1). Detailed definitions, coding, data sources, and timing for all covariates are provided in Supplementary Table S2.

Sociodemographic characteristics included age (categorised as 16–34, 35–49, 50–64, and ≥ 65 years), sex (male or female), ethnicity (White or Non-White), highest educational qualification (no formal qualifications, lower secondary, upper secondary, post-secondary, degree-level qualifications or above, or full-time student), and area-level socioeconomic deprivation measured using quintiles of the 2020 Scottish Index of Multiple Deprivation (SIMD), assigned by residential postcode. Household context variables included: multigenerational household status, defined using CHI- and UPRN-linked household data as households containing at least one person aged ≥ 65 years and at least one co-resident who was 20 or more years younger (ages defined at baseline); and household-level shielding exposure, defined as the presence of at least one other household member recorded on the national shielding list. Health-related covariates included individual-level shielding status, identified from the national shielding register as a binary indicator of clinical extreme vulnerability (Supplementary Table S3), and the presence of other chronic health conditions, derived from 2011 Scottish Census responses and coded as a binary indicator capturing long-term conditions other than the primary disability exposure. COVID-19 vaccination status was obtained from Public Health Scotland national vaccination records and categorised according to the total number of COVID-19 vaccine doses received during follow-up (0, 1, 2, or ≥ 3 doses).

### Statistical Analysis

Cohort characteristics were summarised overall and stratified by disability status using means and standard deviations or medians and interquartile ranges for continuous variables, and counts and percentages for categorical variables.

Crude incidence rates for each COVID-19 outcome were calculated as the number of events divided by total person-years at risk and expressed per 10,000 person-years. Follow-up accrued from 1 March 2020 until the first occurrence of the outcome of interest, death, or 30 April 2022, whichever occurred first. Person-time was calculated separately for each outcome. Associations between disability status and COVID-19 outcomes were examined using Cox proportional hazards regression, with time since cohort entry as the underlying timescale. The comparison group served as the reference category in all models. Hazard ratios (HRs) and 95% confidence intervals (CIs) were estimated separately for infection, hospitalisation, and mortality.

A sequence of models was fitted to examine the impact of stepwise adjustment. Model 1 was unadjusted. Model 2 adjusted for age and sex (demographic factors). Model 3 further adjusted for ethnicity, area-level deprivation, highest educational qualification, and multigenerational household status (socioeconomic factors). Models 2 and 3 primarily adjust for baseline confounders that may influence both disability status and COVID-19 outcomes. Model 4 further adjusted for individual- and household-level shielding, and Model 5 additionally adjusted for other chronic health conditions (health-related factors). Model 6 additionally adjusted for COVID-19 vaccination status and was considered the fully adjusted model. Health-related factors and vaccination status may act as both confounders and mediators, as they could be influenced by disability status while also affecting COVID-19 risk. Consequently, Models 2–3 provide estimates closer to the total effect of disability on COVID-19 outcomes, as they retain pathways operating through differential health status and vaccination uptake. Models 4–6, by additionally adjusting for these factors, yield conditional estimates that may attenuate or shift depending on the direction of confounding and mediation, and should be interpreted as associations independent of measured health-related factors and vaccination status. Sequentially adjusted estimates were visualised across model specifications to illustrate changes in effect estimates with adjustment. The proportional hazards assumption was tested using scaled Schoenfeld residuals to assess independence from time and was visually examined via log–log survival plots. The diagnostics suggested no meaningful violation of the proportional hazards assumption.

Two secondary analyses and one additional sensitivity analysis were conducted to assess the robustness of the main findings. First, follow-up was stratified by testing period to address the potential for differential ascertainment of SARS-CoV-2 infection during the early pandemic when community testing was restricted. Period 1 was defined as 1 March to 27 May 2020, corresponding to the period before the launch of Scotland’s Test and Protect programme. Period 2 was defined as 28 May 2020 to 30 April 2022, corresponding to the period after Test and Protect was introduced, during which symptomatic community testing, contact tracing, and testing capacity were progressively expanded. The fully adjusted model (Model 6) was re-estimated separately within each period for first SARS-CoV-2 infection, COVID-19 hospitalisation, and COVID-19 mortality. Vaccination status was not included as a covariate in Period 1 analyses because COVID-19 vaccines were not yet available to the general adult population during this period; Period 2 analyses retained the same adjustment set as Model 6 in the main analysis. For Period 2 analyses, individuals who had already experienced the relevant outcome in Period 1 were excluded from the risk set, so that Period 2 estimates reflected the association among those still at risk at 28 May 2020. Second, to address possible age-dependent exposure misclassification arising from the 9-year interval between Census disability measurement (2011) and cohort entry (2020), Model 6 was re-estimated separately within each of the four age bands (16–34, 35–49, 50–64, and ≥ 65 years) for all three outcomes. As an additional sensitivity analysis, Model 6 was re-estimated after excluding intellectually disabled participants who reported upper secondary, post-secondary, or university-level qualifications, to assess robustness to potential exposure misclassification within the intellectual disability group.

All analyses were conducted within Scotland’s national secure data environment using Stata/SE 16, and outputs were subject to statistical disclosure control prior to release.

## Results

### Cohort Characteristics

Table 1 presents baseline characteristics of the 3,719,651 adults in the cohort stratified by disability status. Participants with intellectual disability (*n* = 17,354) were slightly younger than the comparison group but markedly younger than those with physical disability, with a mean age of 43.2 years (SD 17.4) compared with 48.5 years (SD 18.6) and 68.1 years (SD 16.2), respectively. Age distribution differed substantially across groups: more than one third of the intellectual disability group were aged 16–34 years (38.2%), whereas nearly two thirds of the physical disability group were aged ≥ 65 years (64.0%); the corresponding proportion in the comparison group was 21.8%. Males comprised a larger proportion of the intellectual disability group (57.8%) than of the physical disability group (49.8%) or the comparison group (47.6%).

Socioeconomic disadvantage was considerably higher among disabled people (physical or intellectual) compared with the comparison population, with the greatest burden borne by individuals with intellectual disabilities. In the most deprived SIMD quintile, 31.2% of the intellectual disability group and 24.2% of the physical disability group were represented, compared with 18.0% in the comparison group. Conversely, representation in the least deprived quintile was substantially lower among disabled participants (9.4% intellectual disability; 14.7% physical disability) than in the comparison population (21.0%). Participants with intellectual disability were slightly less likely to be from black and other minority ethnic groups (non-white) than the comparison group (2.3% vs. 3.0%), whereas those with physical disability were much less likely to be from such groups (1.2% vs. 3.0%). Overall, the cohort remained predominantly white across all groups (97.0–98.8%). Educational attainment differed markedly by disability status. Participants with intellectual disability were far more likely to have no formal qualifications (66.2%) than those with physical disability (45.4%) and the comparison group (17.5%). In contrast, degree-level qualifications or above were least common among the intellectual disability group (2.0%), intermediate among those with physical disability (18.4%), and highest in the comparison population (24.2%). Adults in both disability groups were substantially more likely to live in multigenerational households, particularly those with intellectual disability; 24.0% for intellectual disability, 18.3% for physical disability, and 10.4% for the comparison population.

Disabled participants, particularly those with intellectual disability, had substantially higher prevalence of chronic health conditions than the comparison population. The co-occurrence of other long-term conditions was highest in the intellectual disability group (60.6%), followed by the physical disability group (45.3%), and lowest in the comparison group (19.4%). This gradient was not fully reflected in individual shielding status, however. While shielding was markedly more common among participants with physical disability (7.3%) than in the comparison group (2.3%), levels were very similar between the intellectual disability group (2.0%) and the comparison population. Household member shielding showed a clearer disability gradient, reported by 6.3% of both disability groups compared with 2.8% in the comparison group. COVID-19 vaccination uptake was high across all groups, with most participants receiving two doses (83.9–87.4%). Receipt of three or more doses was highest among those with physical disability (4.2%), compared with the intellectual disability (2.3%) and comparison groups (2.0%).

**Table 1:** Cohort characteristics stratified by disability status

Characteristic	Intellectual disability (*n* = 17,354)	Physical disability (*n* = 377,706)	Comparison group (*n* = 3,324,591)	All (*N* = 3,719,651)
Age (in years)				
Mean (SD)	43.2 (17.4)	68.1 (16.2)	48.5 (18.6)	50.4 (19.3)
Median (IQR)	42 (28–57)	71 (59–80)	49 (33–62)	51 (34–65)
Age category, n (%)				
16–34	6,629 (38.2)	17,167 (4.6)	914,236 (27.5)	938,032 (25.2)
35–49	4,066 (23.4)	30,185 (8.0)	771,517 (23.2)	805,768 (21.7)
50–64	4,456 (25.7)	88,318 (23.4)	912,627 (27.5)	1,005,401 (27.0)
≥ 65	2,203 (12.7)	242,036 (64.0)	726,211 (21.8)	970,450 (26.1)
Sex, n (%)				
Male	10,033 (57.8)	188,206 (49.8)	1,583,033 (47.6)	1,781,272 (47.9)
Female	7,321 (42.2)	189,500 (50.2)	1,741,558 (52.4)	1,938,379 (52.1)
Area-level deprivation (SIMD), n (%)				
Most deprived (Q1)	5,406 (31.2)	91,333 (24.2)	597,326 (18.0)	694,065 (18.7)
Quintile 2	4,411 (25.4)	86,441 (22.9)	648,828 (19.5)	739,680 (19.9)
Quintile 3	3,505 (20.2)	77,089 (20.4)	673,116 (20.3)	753,710 (20.3)
Quintile 4	2,391 (13.8)	67,079 (17.8)	705,838 (21.1)	775,308 (20.8)
Least deprived (Q5)	1,629 (9.4)	55,483 (14.7)	696,703 (21.0)	753,815 (20.3)
Missing	12 (0.1)	281 (0.1)	2,780 (0.1)	3,073 (0.1)
Ethnicity, n (%)				
White	16,961 (97.7)	373,331 (98.8)	3,225,034 (97.0)	3,615,326 (97.2)
Non-White	393 (2.3)	4,375 (1.2)	99,557 (3.0)	104,325 (2.8)
Highest qualification, n (%)				
No formal qualifications	11,487 (66.2)	171,284 (45.4)	582,687 (17.5)	765,458 (20.6)
Lower secondary	1,990 (11.5)	70,856 (18.8)	739,103 (22.2)	811,949 (21.8)
Upper secondary	283 (1.6)	33,099 (8.8)	455,058 (13.7)	488,440 (13.1)
Post-secondary	285 (1.6)	26,125 (6.9)	319,180 (9.6)	345,590 (9.3)
Degree or above	339 (2.0)	69,495 (18.4)	805,997 (24.2)	875,831 (23.6)
Full-time student	2,970 (17.1)	6,847 (1.8)	422,566 (12.7)	432,383 (11.6)
Household and health characteristics, n (%)				
Multigenerational household (Yes)	4,162 (24.0)	69,203 (18.3)	346,228 (10.4)	419,593 (11.3)
Shielding (individual) (Yes)	340 (2.0)	27,670 (7.3)	75,099 (2.3)	103,109 (2.8)
Other household member shielding (Yes)	1,096 (6.3)	23,936 (6.3)	92,734 (2.8)	117,766 (3.2)
Other chronic conditions (Yes)	10,524 (60.6)	171,075 (45.3)	643,817 (19.4)	825,416 (22.2)
COVID-19 vaccination status, n (%)				
Zero dose (Unvaccinated)	1,319 (7.6)	36,374 (9.6)	314,810 (9.5)	352,503 (9.5)
One dose	467 (2.7)	8,455 (2.2)	80,306 (2.4)	89,228 (2.4)
Two doses	15,173 (87.4)	316,901 (83.9)	2,862,363 (86.1)	3,194,437 (85.9)
Three or more doses	395 (2.3)	15,976 (4.2)	67,112 (2.0)	83,483 (2.2)

SIMD = Scottish Index of Multiple Deprivation; SD = standard deviation; IQR = interquartile range. The “Full-time student” category corresponds to Census 2011 code XX, which captures schoolchildren, full-time students living away from home during term time, and individuals aged under 16 years at the time of the Census. Given the 9-year interval between Census enumeration and cohort entry, a substantial proportion of those coded under this category were of school age or otherwise still in education at the time of the Census. Within the intellectual disability group, this was concentrated in the youngest age band: 2,966 of 6,629 participants aged 16–34 years were in this category (44.7%). Counts in older age groups were fewer than 10 and are therefore not reported separately, in line with statistical disclosure control requirements. COVID-19 vaccination status reflects the total number of vaccine doses received during follow-up, not baseline vaccination status

### Crude Incidence Rates of COVID-19 Outcomes by Disability Status

Table 2 shows crude incidence rates of COVID-19 outcomes by disability status. The cohort contributed 5,172,690 person-years of follow-up for SARS-CoV-2 infection, 7,181,732 person-years for COVID-19 hospitalisation, and 7,914,901 person-years for COVID-19 mortality. The crude rate of laboratory-confirmed infection was highest among adults with intellectual disability (1,686.4 per 10,000 person-years; 95% CI 1,637.3–1,737.1), compared with 689.7 (95% CI 682.7–696.7) among those with physical disability and 574.7 (95% CI 572.5–576.9) in the comparison group. Crude hospitalisation rates were highest in the physical disability group (86.9 per 10,000 person-years; 95% CI 84.6–89.2), followed by the intellectual disability group (65.9; 95% CI 57.6–75.4), and were lowest in the comparison group (26.4; 95% CI 26.1–26.8). A similar pattern was observed for COVID-19 mortality, with the highest crude mortality rate among adults with physical disability (71.7 per 10,000 person-years; 95% CI 69.8–73.6), compared with 30.1 (95% CI 25.0–36.3) among those with intellectual disability and 10.2 (95% CI 10.1–10.5) in the comparison group. Overall, across the full cohort, crude rates were 592.2 per 10,000 person-years (95% CI 590.1–594.3) for infection, 31.9 (95% CI 31.5–32.4) for hospitalisation, and 16.3 (95% CI 16.0–16.6) for mortality (Supplementary Figure S2).

**Table 2:** Crude incidence rates of COVID-19 outcomes by disability status

Outcome	Disability status	Events (*n*)	Rate per 10,000 person-years	95% CI
COVID-19 infection	Intellectual disability	4,388	1,686.4	1,637.3–1,737.1
	Physical disability	36,992	689.7	682.7–696.7
	Comparison group	264,963	574.7	572.5–576.9
	All	306,343	592.2	590.1–594.3
COVID-19 hospitalisation	Intellectual disability	213	65.9	57.6–75.4
	Physical disability	5,494	86.9	84.6–89.2
	Comparison group	17,238	26.4	26.1–26.8
	All	22,945	31.9	31.5–32.4
COVID-19 mortality	Intellectual disability	110	30.1	25.0–36.3
	Physical disability	5,499	71.7	69.8–73.6
	Comparison group	7,284	10.2	10.1–10.5
	All	12,893	16.3	16.0–16.6

Crude incidence rates are expressed per 10,000 person-years of follow-up, with 95% confidence intervals estimated assuming a Poisson distribution. Outcomes were ascertained between 1 March 2020 and 30 April 2022. Person-time accrued from 1 March 2020 until the first occurrence of the outcome of interest, death, or end of follow-up, whichever occurred first, and was calculated separately for each outcome

### Associations Between Disability Status and COVID-19 Outcomes

Disabled participants experienced elevated risks of COVID-19 infection, hospitalisation, and mortality compared with the comparison group, with the highest risks observed among individuals with intellectual disability (Table 3). Fully adjusted associations for disability status and all covariates included in the final models are provided in Supplementary Table S4.

**Table 3:** Crude and sequentially adjusted Cox proportional hazards models examining associations between disability status and COVID-19 infection, hospitalisation, and mortality

Model	Disability status	COVID-19 infection HR (95% CI), *p*-value	COVID-19 hospitalisation HR (95% CI), *p*-value	COVID-19 mortality HR (95% CI), *p*-value
Model 1: Crude (unadjusted)	Comparison group	Reference	Reference	Reference
	Intellectual disability	3.03 (2.94–3.12), *p* < 0.001	1.82 (1.59–2.09), *p* < 0.001	1.57 (1.30–1.89), *p* < 0.001
	Physical disability	1.22 (1.21–1.24), *p* < 0.001	1.46 (1.42–1.51), *p* < 0.001	1.57 (1.51–1.62), *p* < 0.001
Model 2: + Age and sex	Comparison group	Reference	Reference	Reference
	Intellectual disability	2.69 (2.61–2.77), *p* < 0.001	1.92 (1.68–2.21), *p* < 0.001	2.02 (1.67–2.44), *p* < 0.001
	Physical disability	1.62 (1.60–1.64), *p* < 0.001	1.31 (1.27–1.35), *p* < 0.001	1.34 (1.29–1.39), *p* < 0.001
Model 3: + Ethnicity, deprivation, qualification, and multigenerational household	Comparison group	Reference	Reference	Reference
	Intellectual disability	2.51 (2.44–2.59), *p* < 0.001	1.57 (1.37–1.80), *p* < 0.001	1.63 (1.35–1.98), *p* < 0.001
	Physical disability	1.56 (1.55–1.58), *p* < 0.001	1.23 (1.19–1.27), *p* < 0.001	1.27 (1.22–1.32), *p* < 0.001
Model 4: + Individual and household shielding	Comparison group	Reference	Reference	Reference
	Intellectual disability	2.51 (2.43–2.59), *p* < 0.001	1.57 (1.37–1.80), *p* < 0.001	1.59 (1.32–1.92), *p* < 0.001
	Physical disability	1.56 (1.54–1.58), *p* < 0.001	1.21 (1.17–1.25), *p* < 0.001	1.25 (1.20–1.29), *p* < 0.001
Model 5: + Other chronic health conditions	Comparison group	Reference	Reference	Reference
	Intellectual disability	2.66 (2.58–2.74), *p* < 0.001	1.53 (1.34–1.76), *p* < 0.001	1.55 (1.28–1.87), *p* < 0.001
	Physical disability	1.60 (1.58–1.62), *p* < 0.001	1.20 (1.16–1.24), *p* < 0.001	1.23 (1.19–1.28), *p* < 0.001
Model 6: Final model (+ vaccination status)	Comparison group	Reference	Reference	Reference
	Intellectual disability	2.65 (2.57–2.73), *p* < 0.001	1.60 (1.40–1.83), *p* < 0.001	1.58 (1.30–1.91), *p* < 0.001
	Physical disability	1.60 (1.58–1.62), *p* < 0.001	1.16 (1.12–1.19), *p* < 0.001	1.23 (1.19–1.28), *p* < 0.001

Hazard ratios (HRs) and 95% confidence intervals (CIs) were estimated using Cox proportional hazards regression. Adults without recorded intellectual or physical disability were used as the reference category in all models. Follow-up commenced on 1 March 2020 and continued until the first occurrence of the outcome of interest, death, or 30 April 2022, whichever occurred first. Sequential models were fitted to examine the contribution of demographic, socioeconomic, health-related, and vaccination factors. Model 1 was unadjusted. Model 2 adjusted for demographic characteristics (age and sex). Model 3 additionally adjusted for socioeconomic and ethnic factors, including ethnicity, area-level deprivation, highest educational qualification, and multigenerational household status. Model 4 further adjusted for individual- and household-level shielding status. Model 5 additionally adjusted for the presence of other chronic health conditions. Model 6 additionally adjusted for COVID-19 vaccination status

In crude analyses (Model 1), participants with intellectual disability had threefold higher risk of COVID-19 infection (HR 3.03, 95% CI 2.94–3.12), alongside increased risks of hospitalisation (HR 1.82, 95% CI 1.59–2.09) and mortality (HR 1.57, 95% CI 1.30–1.89). Risks were also elevated among those with physical disability, although effect sizes were smaller for infection (HR 1.22, 95% CI 1.21–1.24) and hospitalisation (HR 1.46, 95% CI 1.42–1.51) but comparable for mortality (HR 1.57, 95% CI 1.51–1.62).

Adjustment for age and sex attenuated infection risk (Model 2) among participants with intellectual disability (HR 2.69, 95% CI 2.61–2.77) but increased relative hazards for hospitalisation (HR 1.92, 95% CI 1.68–2.21) and mortality (HR 2.02, 95% CI 1.67–2.44), reflecting the younger age profile of this group. In contrast, for physical disability, age–sex adjustment increased infection risk (HR 1.62, 95% CI 1.60–1.64) while reducing hospitalisation and mortality hazards, consistent with their markedly older baseline age structure.

Further adjustment for ethnicity, area deprivation, educational attainment, and multigenerational household composition (Model 3) modestly attenuated risks but did not eliminate disparities. Intellectual disability remained associated with elevated infection (HR 2.51, 95% CI 2.44–2.59), hospitalisation (HR 1.57, 95% CI 1.37–1.80), and mortality (HR 1.63, 95% CI 1.35–1.98). Corresponding estimates for physical disability were infection (HR 1.56, 95% CI 1.55–1.58), hospitalisation (HR 1.23, 95% CI 1.19–1.27), and mortality (HR 1.27, 95% CI 1.22–1.32).

Additional adjustment for individual and household shielding (Model 4) and other chronic health conditions (Model 5) led to modest attenuation in hospitalisation and mortality risks in both disability groups, although excess risk persisted. For intellectual disability, hazard ratios remained elevated for infection (HR 2.66, 95% CI 2.58–2.74), hospitalisation (HR 1.53, 95% CI 1.34–1.76), and mortality (HR 1.55, 95% CI 1.28–1.87). Estimates for physical disability were infection (HR 1.60, 95% CI 1.58–1.62), hospitalisation (HR 1.20, 95% CI 1.16–1.24), and mortality (HR 1.23, 95% CI 1.19–1.28), respectively.

**Fig. 2:**
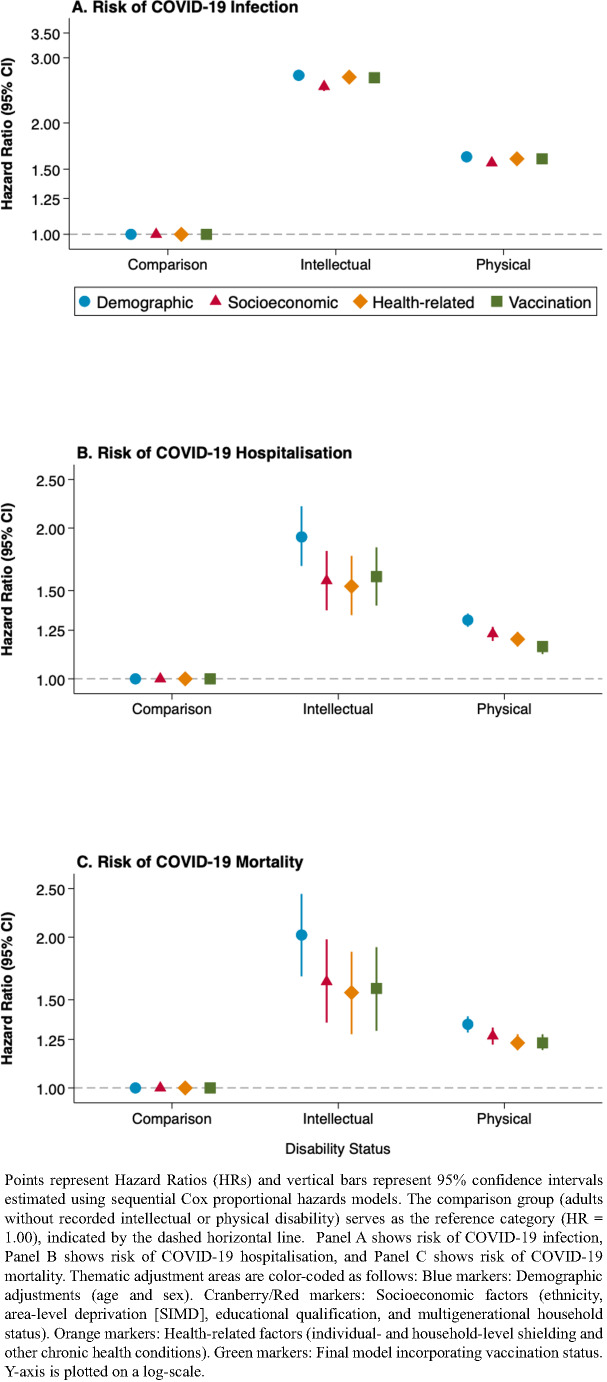
Stepwise adjusted hazard ratios for COVID-19 infection, hospitalisation, and mortality by disability status

In the fully adjusted model incorporating vaccination status (Model 6), elevated risks persisted. Intellectual disability remained associated with substantially higher infection risk (HR 2.65, 95% CI 2.57–2.73), as well as increased hospitalisation (HR 1.60, 95% CI 1.40–1.83) and mortality (HR 1.58, 95% CI 1.30–1.91). Physical disability was also associated with increased infection (HR 1.60, 95% CI 1.58–1.62), hospitalisation (HR 1.16, 95% CI 1.12–1.19), and mortality (HR 1.23, 95% CI 1.19–1.28), although effect sizes were consistently smaller than those observed for intellectual disability (Fig. 2).

### Secondary Analyses

When follow-up was stratified by testing period (Table 4), elevated hazards persisted in Period 2, when community testing was widely available. For first SARS-CoV-2 infection, the adjusted hazard ratio for intellectual disability attenuated from 3.85 (95% CI 3.41–4.36) in Period 1 to 2.56 (95% CI 2.48–2.64) in Period 2, and the corresponding hazard ratio for physical disability changed from 1.69 (95% CI 1.62–1.77) to 1.56 (95% CI 1.54–1.58). This pattern is consistent with some contribution of differential ascertainment in the early restricted-testing period, but with substantially elevated infection risks remaining in the broader-testing period when detection was more complete. For COVID-19 hospitalisation and mortality, hazard ratios were similar or slightly higher in Period 2 than in Period 1 across both disability groups, indicating that elevated risks of severe outcomes are not artefacts of early-period testing constraints.

**Table 4:** Associations between disability status and COVID-19 outcomes, stratified by testing period

Outcome	Disability status	Period 1: 1 Mar – 27 May 2020 Adjusted HR (95% CI), *p*-value	Period 2: 28 May 2020–30 Apr 2022 Adjusted HR (95% CI), *p*-value
COVID-19 infection	Comparison group	Reference	Reference
	Intellectual disability	3.85 (3.41–4.36), *p* < 0.001	2.56 (2.48–2.64), *p* < 0.001
	Physical disability	1.69 (1.62–1.77), *p* < 0.001	1.56 (1.54–1.58), *p* < 0.001
COVID-19 hospitalisation	Comparison group	Reference	Reference
	Intellectual disability	1.49 (1.11–2.02), *p* = 0.009	1.62 (1.39–1.88), *p* < 0.001
	Physical disability	1.12 (1.04–1.20), *p* = 0.001	1.17 (1.12–1.21), *p* < 0.001
COVID-19 mortality	Comparison group	Reference	Reference
	Intellectual disability	1.35 (0.96–1.89), *p* = 0.085	1.59 (1.26–2.00), *p* < 0.001
	Physical disability	1.11 (1.04–1.19), *p* = 0.003	1.29 (1.23–1.34), *p* < 0.001

Period 1 was defined as 1 March 2020 to 27 May 2020, corresponding to the earlier phase of the pandemic when testing was more restricted. Period 2 was defined as 28 May 2020 to 30 April 2022, corresponding to the period following the introduction of Scotland’s Test and Protect programme and broader community testing. Hazard ratios were estimated using Cox proportional hazards models adjusted for age, sex, ethnicity, area deprivation, highest qualification, multigenerational household status, individual shielding, household shielding, other chronic conditions, and vaccination status. Vaccination status was included as a covariate in Period 2 only, as COVID-19 vaccines were not yet available during Period 1. For Period 2, individuals who had already experienced the relevant outcome in Period 1 were excluded from the risk set

In age-stratified analyses (Table 5), elevated hazards of first SARS-CoV-2 infection were observed in every age band for both disability groups, with the disability gradient strengthening with age. The hazard ratio for intellectual disability rose from 2.20 (95% CI 2.09–2.32) in those aged 16–34 years to 5.14 (95% CI 4.73–5.57) in those aged 65 years and over, and the hazard ratio for physical disability rose from 1.31 (95% CI 1.26–1.36) to 1.85 (95% CI 1.82–1.88) across the same age range. For COVID-19 hospitalisation, intellectual disability was associated with elevated hazards across all four age bands (hazard ratios ranging from 1.52 to 1.74), whereas estimates for physical disability were close to or below the null in the three younger age bands and elevated only in those aged 65 years and over. For COVID-19 mortality, associations with intellectual disability were elevated in the 35–49 (HR 3.71, 95% CI 1.88–7.31) and 50–64 (HR 1.67, 95% CI 1.21–2.31) age bands, with a high point estimate in those aged 16–34 years that was statistically imprecise owing to small numbers (*n* = 42 deaths), and attenuated in those aged 65 years and over; physical disability was most clearly associated with mortality in older adults. These patterns, particularly for infection and hospitalisation, are not consistent with the main associations being driven primarily by incomplete capture of disability status among younger cohort members in the 2011 Census.

**Table 5:** Associations between disability status and COVID-19 outcomes, by age group

Outcome	Disability status	16–34 years HR (95% CI), *p*-value	35–49 years HR (95% CI), *p*-value	50–64 years HR (95% CI), *p*-value	≥ 65 years HR (95% CI), *p*-value
COVID-19 infection	Comparison group	Reference	Reference	Reference	Reference
	Intellectual disability	2.20 (2.09–2.32), *p* < 0.001	2.42 (2.26–2.58), *p* < 0.001	3.01 (2.84–3.20), *p* < 0.001	5.14 (4.73–5.57), *p* < 0.001
	Physical disability	1.31 (1.26–1.36), *p* < 0.001	1.31 (1.26–1.35), *p* < 0.001	1.37 (1.34–1.40), *p* < 0.001	1.85 (1.82–1.88), *p* < 0.001
COVID-19 hospitalisation	Comparison group	Reference	Reference	Reference	Reference
	Intellectual disability	1.60 (1.09–2.33), *p* = 0.016	1.52 (1.09–2.12), *p* = 0.014	1.74 (1.40–2.17), *p* < 0.001	1.58 (1.23–2.03), *p* < 0.001
	Physical disability	1.01 (0.77–1.32), *p* = 0.928	0.84 (0.72–0.98), *p* = 0.028	1.03 (0.96–1.11), *p* = 0.463	1.21 (1.16–1.26), *p* < 0.001
COVID-19 mortality	Comparison group	Reference	Reference	Reference	Reference
	Intellectual disability	3.09 (0.75–12.70), *p* = 0.118	3.71 (1.88–7.31), *p* < 0.001	1.67 (1.21–2.31), *p* = 0.002	1.13 (0.86–1.47), *p* = 0.376
	Physical disability	1.34 (0.43–4.19), *p* = 0.613	1.21 (0.82–1.77), *p* = 0.331	1.14 (1.00–1.31), *p* = 0.057	1.25 (1.20–1.30), *p* < 0.001

Abbreviations: HR, hazard ratio; CI, confidence interval. Hazard ratios were estimated using Cox proportional hazards models adjusted for sex, ethnicity, area deprivation, highest qualification, multigenerational household status, individual shielding, household shielding, other chronic conditions, and COVID-19 vaccination status

In a further sensitivity analysis presented in Supplementary Table S5, hazard ratios were re-estimated after excluding the 907 intellectually disabled participants (5.2% of the intellectual disability group) who reported upper secondary, post-secondary, or university-level qualifications. Hazard ratios were essentially unchanged across all three outcomes, indicating that the observed associations are robust to potential exposure misclassification arising from the small proportion of the intellectual disability group with higher educational attainment.

## Discussion

Our analyses show that both intellectual disability and physical disability were associated with higher hazards of COVID-19 infection, hospitalisation, and mortality compared with adults without recorded disability. Although this aligns with prior UK and international evidence [11], the present study extends the literature by assessing distinct disability types and multiple COVID-19 outcomes within a single national cohort, using a shared comparison group. This design highlights meaningful heterogeneity: risks were consistently more pronounced for intellectual disability, while physical disability showed more moderate elevations that varied across the disease pathway. The fact that disparities were observed for infection as well as severe outcomes indicates that disability-related inequalities operate from exposure and acquisition through to clinical progression. 

Interpretation of the sequential models requires care, particularly in distinguishing confounding from mediation. Models adjusting for demographic and socioeconomic factors primarily account for baseline differences that shape both disability status and COVID-19 risk. The persistence of associations after these adjustments indicates that inequalities are not fully explained by population structure or socioeconomic circumstances. In contrast, models that additionally adjust for shielding, chronic conditions, and vaccination introduce variables that may be both confounders and intermediates. Chronic morbidity and shielding eligibility can reflect pre-existing health risk, but they may also be influenced by disability-related biological, behavioural, and structural pathways, and shielding guidance in the UK explicitly incorporated disability-related risk [20]. Vaccination uptake and timing may also differ by disability status because of prioritisation, access barriers, and support needs [21, 22]. As a result, later models may estimate associations conditional on these mechanisms rather than the total burden attributable to disability [23]. The modest attenuation after including health-related factors and vaccination suggests partial explanation through differential health status and vaccine-mediated protection, but remaining elevations point to additional vulnerability not captured by measured pathways. 

For adults with intellectual disability, crude infection burden was markedly elevated and increased hazards were evident across infection, hospitalisation, and mortality in all modelling stages. These findings are consistent with evidence from OpenSAFELY and earlier Scotland-based studies documenting excess COVID-19 risks among people with intellectual disabilities [12, 13]. Attenuation after adjustment for ethnicity, deprivation, education, and household composition indicates that social patterning contributes meaningfully to disparities, but residual associations imply that conventional sociodemographic explanations are insufficient on their own. We interpret this as reflecting convergent influences operating across multiple levels. Higher infection risk may plausibly arise from communal living, reliance on close-contact support from paid carers or support workers, and challenges maintaining distancing or infection control [5, 6, 14]. Persistently elevated severe outcomes likely reflect both higher prevalence of conditions associated with severe COVID-19—such as epilepsy, respiratory impairment, and immunological dysregulation [24–26]—and barriers to timely recognition and escalation of care, including communication difficulties, diagnostic overshadowing, and delays in clinical assessment [5]. Continued excess mortality after accounting for recorded comorbidity suggests that administrative health records may incompletely capture disability-specific clinical vulnerability, and/or that inequities in access and quality of care persist despite formal risk stratification. The ascertained prevalence of intellectual disability in the cohort (0.5%) is lower than the expected population prevalence of approximately 2–3% for children and younger adults [16], reflecting recognised under-capture of milder cases by Census-based ascertainment. People with milder intellectual disability who were not classified as such in the Census would have been included in the comparison group, biasing the reported hazard ratios toward the null and meaning the elevated risks reported here are likely conservative estimates. 

For physical disability, crude hospitalisation and mortality rates were substantially higher than in the comparison group, but interpretation of unadjusted estimates is shaped by strong age patterning. The increase in infection hazards after accounting for age and sex is consistent with negative confounding, where older age structure suppresses crude infection risk because older adults experienced lower exposure during parts of the pandemic due to shielding, reduced social contact, and earlier vaccine prioritisation [27]. Once age is accounted for, the underlying positive association between physical disability and infection becomes clearer. Socioeconomic adjustment also produced further attenuation, indicating that material disadvantage and constrained resources contribute to excess risk, consistent with wider evidence linking disability to socioeconomic disadvantage [28, 29]. Additional attenuation after adjusting for chronic conditions and vaccination is consistent with the close coupling of physical disability, multimorbidity, and age-related health decline, which are established predictors of severe COVID-19 [30–32]. Nonetheless, persistent associations in fully adjusted models indicate independent vulnerability beyond measured demographic, socioeconomic, and clinical factors. 

The secondary analyses presented strengthen the interpretation of the main findings. Stratification by testing period showed that, although infection hazards in the early restricted-testing period were higher than in the broader-testing period, substantial elevated risks of infection persisted in Period 2, when detection was more complete, and hazards for hospitalisation and mortality were similar or slightly higher in Period 2 than in Period 1. Age-stratified analyses indicated that elevated hazards of infection were observed across all age bands for both disability groups, with the disability gradient strengthening with age; elevated hazards of hospitalisation in the intellectual disability group were observed across all age bands; and for mortality, elevated hazards were most evident in middle age bands for intellectual disability and in older adults for physical disability. Together, these patterns suggest that the observed associations between disability status and COVID-19 outcomes are unlikely to be explained primarily by differential ascertainment in the early pandemic or by age-dependent exposure misclassification arising from the use of 2011 Census disability measures, supporting the robustness of the main estimates. 

These findings support the explicit inclusion of disability status, particularly intellectual disability, in pandemic preparedness and clinical risk stratification frameworks alongside established markers such as age and multimorbidity. The pattern of inequalities across infection and severe outcomes underscores the need for integrated prevention strategies, including rapid access to testing, proactive clinical assessment following suspected infection, and tailored follow-up responsive to communication needs and support arrangements. Vaccination programmes and public health interventions should be designed to be accessible and inclusive, incorporating outreach, information in accessible formats, flexible delivery for those with mobility or communication requirements, and training for healthcare staff on reasonable adjustments. In practice, reducing inequalities will also require strengthened infection prevention and control in residential and supported living settings where close-contact care is provided, improved accessible public health communication, and adequate training, resources, and protective equipment for carers and support workers. 

More broadly, preventing avoidable disparities in future respiratory epidemics will require systematic removal of structural barriers to equitable care [33–35]. This includes routine provision of communication support, embedding reasonable adjustments into clinical pathways, addressing diagnostic overshadowing and implicit bias that may delay recognition of acute illness, and ensuring triage and resource allocation processes do not inadvertently disadvantage disabled people. Ultimately, achieving equity for disabled populations during public health emergencies requires not only clinical risk stratification but sustained commitments to accessible services, inclusive policy design, and equitable healthcare delivery. 

This study benefits from a large, population-wide cohort of 3.7 million adults, enabling precise estimation of associations across multiple outcomes with narrow confidence intervals. The use of national surveillance and administrative datasets supported consistent recording of detected infection, hospitalisation, and mortality events over a long follow-up period, though this should not be equated with consistent ascertainment of underlying SARS-CoV-2 infection, which depended on testing access and uptake. Separating intellectual disability from physical disability provides more policy-relevant evidence than studies that treat disability as a single exposure category. The sequential adjustment strategy improves transparency by showing how effect estimates change with the addition of sociodemographic, household, health, and vaccination-related factors. The breadth of covariates available in linked data strengthens internal validity by reducing confounding from major demographic and socioeconomic determinants of COVID-19 outcomes. 

Several limitations should be considered when interpreting these findings. First, disability status was derived from self-reported long-term conditions recorded in the 2011 Scottish Census and may not fully reflect disability status during 2020–2022, introducing the possibility of misclassification. This is likely to be age-dependent: among younger adults, disability may have been incompletely captured at Census, while among older adults, physical disability status may have changed over time. As shown in the age-stratified analyses, this concern is unlikely to fully explain the observed associations, particularly for intellectual disability; however, weaker estimates for physical disability in younger age bands may partly reflect attenuation due to physical disability acquired between Census enumeration and cohort entry, which would have biased the contrast toward the null. Second, some potentially important determinants of risk were unavailable or not fully captured, including disability severity, functional limitations, care intensity, occupational exposure, and certain disability-specific clinical factors such as Down syndrome. 

Third, ascertainment of infection depended on testing access and behaviour, both of which changed substantially over time and may have differed by disability status. Community testing was more restricted before Scotland’s Test and Protect programme was introduced on 28 May 2020 and became broader thereafter, including priority testing in high-risk settings such as care homes and hospitals. As shown in the secondary analyses, elevated hazards persisted in the broader-testing period; the infection outcome should nonetheless be interpreted as first recorded or detected infection rather than necessarily true incident infection. Fourth, residence type was not directly modelled. Some of the elevated risk observed, particularly for intellectual disability, may therefore reflect pathways operating through communal or supported living arrangements, which could act as mediators of disability-related risk and exposure rather than simply as unmeasured confounders. 

Fifth, although other chronic conditions were measured only as a binary Census-derived indicator, adjustment for age, sex, area deprivation, and shielding status is likely to have captured some additional confounding related to underlying health risk. However, more detailed information on comorbidity type, number, and severity was unavailable, so residual confounding cannot be excluded, particularly for hospitalisation and mortality. Sixth, vaccination status was operationalised as a categorical measure and may not have fully captured the timing of doses relative to events. Seventh, ethnicity was measured using broad categories, with small numbers in some groups, limiting assessment of heterogeneity and leaving some potential for residual confounding. 

## Conclusion

In this population-wide cohort of adults in Scotland, both intellectual disability and physical disability were associated with higher risks of COVID-19 infection, hospitalisation, and mortality compared with adults without recorded disability. The magnitude and pattern of risk differed by disability type, with intellectual disability showing particularly elevated relative risks across outcomes and physical disability exhibiting a substantial burden of severe outcomes. These associations persisted after extensive adjustment for demographic, socioeconomic, health-related, and vaccination factors, indicating that measured differences in population structure and clinical vulnerability do not fully explain the observed disparities. The findings underline disability status as an important marker of vulnerability across the COVID-19 disease pathway, rather than risk being confined to a single outcome or stage. 

By distinguishing intellectual disability from physical disability and examining infection, hospitalisation, and mortality within the same national cohort, this study provides robust, Scotland-specific evidence on disability-related inequalities during the COVID-19 pandemic. The results highlight the need for pandemic preparedness and response strategies that explicitly account for disability, alongside age and comorbidity, to reduce avoidable harm. Addressing structural barriers to care, ensuring accessible preventive measures, and supporting those who provide close-contact care will be central to mitigating risk. More broadly, these findings reinforce the importance of inclusive public health planning that recognises the heterogeneity of disabled populations. Incorporating disability-sensitive approaches into future respiratory epidemic responses is essential to achieving equitable health outcomes. 

## Supplementary Information

Below is the link to the electronic supplementary material.

Supplementary Material 1 (PDF 449 KB)

## Data Availability

Data may be obtained from a third party and are not publicly available.
