# Supplementary material for: Intellectual and Physical Disability and Risk of COVID-19 Infection, Hospitalisation, and Mortality: A National Cohort of 3.7 Million Adults in Scotland
Source: J Epidemiol Glob Health. 2026 May 21;16(1):93. doi: 10.1007/s44197-026-00581-4 (PMC13391471; doi:10.1007/s44197-026-00581-4)
Supplement: Supplementary file 1 — Supplementary Material 1 (PDF 449 KB) [file 44197_2026_581_MOESM1_ESM.pdf]

Intellectual and Physical Disability and Risk of COVID-19 Infection, Hospitalisation, and Mortality: A National Cohort of 3.7 Million Adults in Scotland

Supplementary Table S1. Operational definitions and data sources for COVID-19 outcomes (Scotland; follow-up 1 March 2020–30 April 2022)

Outcome	Data source(s)	Operational definition used in this study	Key derivation notes / reporting context
Laboratory-confirmed SARS-CoV-2 infection (first event)	Public Health Scotland (PHS) COVID-19 testing data within the PHS COVID-19 Research Database	First recorded positive SARS-CoV-2 test during follow-up (laboratory-confirmed). Each person contributes at most one infection event (first positive).	We define infection as first positive test only (not reinfections), so estimates reflect first detected infection during follow-up rather than all episodes.
COVID-19 hospitalisation (first event)	Scottish Morbidity Record 01 (SMR01) linked to testing data	First qualifying inpatient admission during follow-up meeting either: (a) COVID-19 recorded as primary or secondary diagnosis using ICD-10 U07.1 (confirmed COVID-19) or U07.2 (suspected COVID-19) in any diagnosis position; or (b) admission occurring within 14 days of a laboratory-confirmed positive SARS-CoV-2 test. Each person contributes at most one hospitalisation event.	This hybrid approach (diagnosis-based and test-linked admissions) is consistent with methods used in Scottish linked-data studies defining COVID-related hospitalisation using U07.1/U07.2 and/or linkage to recent positive tests.
COVID-19-related mortality	National Records of Scotland (NRS) death registrations linked to testing data	Death classified as COVID-19-related if either (a) COVID-19 recorded on the death certificate as underlying or contributory cause, or (b) death occurred within 28 days of a laboratory-confirmed SARS-CoV-2 infection.	In Scotland, COVID-19 mortality is routinely reported using two complementary definitions: deaths occurring within 28 days of a laboratory-confirmed SARS-CoV-2 infection and deaths where COVID-19 is recorded on the death certificate as an underlying or contributory cause. In this study, we applied a transparent combined definition, classifying a death as COVID-19–related if either criterion was met.

Source: Scottish Government: Coronavirus (COVID-19) data: definitions and sources. https://www.gov.scot/publications/coronavirus-covid-19-data-definitions-and-sources/

Supplementary Table S2. Covariates included in the analysis: definitions, data sources, and coding

Covariate domain	Variable	Data source	Definition and coding	Timing
**Demographic/Socioeconomic**	Age	Community Health Index (CHI)	Age at cohort index date (1 March 2020), modelled categorically as 16–34, 35–49, 50–64, and ≥65 years	Baseline
	Sex	CHI	Male; Female	Baseline
	Ethnicity	2011 Scottish Census	Self-reported ethnicity, categorised as White or Non-White	2011 Census (used as proxy for baseline 2020)
	Highest educational qualification	2011 Scottish Census	Six-category variable derived from Census 2011 qualifications question. No formal qualifications = Census code 20 (no qualifications); lower secondary = Level 1; upper secondary = Level 2; post-secondary = Level 3; degree or above = Level 4 and above; full-time student = code XX (schoolchildren, full-time students living away from home during term time, and all individuals aged under 16 years at the time of the Census enumeration).	2011 Census (used as proxy for baseline 2020)
	Area-level socioeconomic deprivation	SIMD 2020	Quintiles of the Scottish Index of Multiple Deprivation (1 = most deprived; 5 = least deprived), assigned by residential postcode	Baseline
**Household context**	Multigenerational household	CHI register + OS UPRN	UPRN-linked households containing ≥1 person aged ≥65 years and ≥1 co-resident at least 20 years younger (ages at baseline)	Baseline
	Household-level shielding	National shielding register + OS UPRN	Binary indicator for presence of ≥1 other UPRN-linked household member recorded on the national shielding list	Baseline
**Health-related**	Individual shielding status	National shielding register	Binary indicator identifying individuals recorded on the shielding list as clinically extremely vulnerable	Baseline
	Other chronic health conditions	2011 Scottish Census	Binary indicator capturing the presence of long-term health conditions other than the primary disability exposure, including mental health conditions, long-term illness, developmental disorders, or other chronic conditions	2011 Census (used as proxy for baseline 2020)
**COVID-19 response**	Vaccination status	Public Health Scotland national vaccination records	Categorised according to the total number of COVID-19 vaccine doses received during follow-up (0, 1, 2, or ≥3 doses)	During follow-up

Table S3. Description of the groups of people and conditions included in Scotland's COVID-19 Highest Risk (Shielding) List

No.	Group
1	Solid organ transplant recipients who remain on long-term immune suppression therapy
2	People with specific cancers (e.g. lung cancer) and receiving treatments
3	People with severe respiratory conditions including all people with cystic fibrosis, severe asthma and severe chronic obstructive pulmonary disease (COPD)
4	People with rare diseases and inborn errors of metabolism that significantly increase the risk of infections (such as severe combined immunodeficiency and homozygous sickle cell)
5	People on immunosuppression therapies sufficient to significantly increase risk of infection
6	People who are pregnant with significant heart disease
7	People on home oxygen
8	People with severe bronchiectasis and pulmonary hypertension
9	People who have had their spleen removed
10	People on renal dialysis
11	People with Down Syndrome
12	People with chronic kidney disease
13	People added to the Highest Risk List based on clinician judgement

Source: Scottish Government. *Coronavirus (COVID-19) advice for people on the Highest Risk List: evidence review.* Published 27 April 2022. https://www.gov.scot/publications/review-evidence-scottish-government-advice-people-scotlands-highest-risk-list/pages/4/

Table S4. Fully adjusted associations between disability status and COVID-19 infection, hospitalisation, and mortality, including all model covariates

Covariate	Category	COVID-19 infection HR (95% CI), *p-value*	COVID-19 hospitalisation HR (95% CI), *p-value*	COVID-19 mortality HR (95% CI), *p-value*
**Disability status**				
	Comparison group	Reference	Reference	Reference
	Intellectual disability	2.65 (2.57–2.73), *p*<0.001	1.60 (1.40–1.83), *p*<0.001	1.58 (1.30–1.91), *p*<0.001
	Physical disability	1.60 (1.58–1.62), *p*<0.001	1.16 (1.12–1.19), *p*<0.001	1.23 (1.19–1.28), *p*<0.001
**Age group (years)**				
	16–34	Reference	Reference	Reference
	35–49	0.95 (0.94–0.96), *p*<0.001	1.59 (1.49–1.70), *p*<0.001	5.06 (3.48–7.35), *p*<0.001
	50–64	0.79 (0.78–0.80), *p*<0.001	1.99 (1.87–2.11), *p*<0.001	18.60 (13.0–26.6), *p*<0.001
	≥65	0.51 (0.51–0.52), *p*<0.001	2.06 (1.93–2.19), *p*<0.001	45.50 (31.9–64.8), *p*<0.001
**Sex**				
	Male	Reference	Reference	Reference
	Female	0.94 (0.93–0.95), *p*<0.001	0.85 (0.83–0.88), *p*<0.001	0.81 (0.78–0.84), *p*<0.001
**Ethnicity**				
	Non-White	Reference	Reference	Reference
	White	0.89 (0.87–0.91), *p*<0.001	0.44 (0.41–0.47), *p*<0.001	0.65 (0.56–0.74), *p*<0.001
**Area-level deprivation (SIMD)**				
	Most deprived	Reference	Reference	Reference
	Quintile 2	0.94 (0.93–0.95), *p*<0.001	0.88 (0.85–0.91), *p*<0.001	0.91 (0.87–0.96), *p*<0.001
	Quintile 3	0.85 (0.84–0.86), *p*<0.001	0.75 (0.71–0.78), *p*<0.001	0.81 (0.77–0.85), *p*<0.001
	Quintile 4	0.85 (0.84–0.86), *p*<0.001	0.71 (0.68–0.73), *p*<0.001	0.81 (0.77–0.86), *p*<0.001
	Least deprived	0.86 (0.85–0.87), *p*<0.001	0.68 (0.65–0.71), *p*<0.001	0.80 (0.75–0.85), *p*<0.001
**Educational qualification**				
	No qualifications	Reference	Reference	Reference
	Lower secondary	1.06 (1.05–1.08), *p*<0.001	0.92 (0.89–0.95), *p*<0.001	0.90 (0.85–0.94), *p*<0.001
	Upper secondary	0.97 (0.95–0.98), *p*<0.001	0.92 (0.87–0.96), *p*<0.001	0.81 (0.75–0.88), *p*<0.001
	Post-secondary	0.96 (0.94–0.97), *p*<0.001	0.89 (0.85–0.94), *p*<0.001	0.82 (0.75–0.89), *p*<0.001
	Degree or above	0.79 (0.78–0.80), *p*<0.001	0.75 (0.72–0.79), *p*<0.001	0.75 (0.71–0.80), *p*<0.001
	Full-time student	1.24 (1.23–1.27), *p*<0.001	0.64 (0.58–0.70), *p*<0.001	0.41 (0.21–0.82), *p*=0.012
**Multigenerational household**				
	No	Reference	Reference	Reference
	Yes	1.08 (1.07–1.10), *p*<0.001	1.17 (1.13–1.21), *p*<0.001	1.23 (1.18–1.29), *p*<0.001
**Individual shielding**				
	No	Reference	Reference	Reference
	Yes	0.99 (0.96–1.01), *p*=0.398	1.27 (1.22–1.34), *p*<0.001	0.94 (0.90–0.99), *p*=0.023
**Household member shielding**				
	No	Reference	Reference	Reference
	Yes	1.27 (1.25–1.30), *p*<0.001	1.41 (1.33–1.48), *p*<0.001	2.10 (2.00–2.21), *p*<0.001
**Other chronic conditions**				

Covariate	Category	COVID-19 infection HR (95% CI), ***p-value***	COVID-19 hospitalisation HR (95% CI), ***p-value***	COVID-19 mortality HR (95% CI), ***p-value***
	No	Reference	Reference	Reference
	Yes	0.87 (0.86–0.88), *p*<0.001	1.08 (1.05–1.11), *p*<0.001	1.17 (1.13–1.21), *p*<0.001
**Vaccination status**				
	Zero doses	Reference	Reference	Reference
	One dose	1.17 (1.14–1.20), *p*<0.001	0.41 (0.38–0.44), *p*<0.001	0.13 (0.12–0.14), *p*<0.001
	Two doses	1.04 (1.03–1.06), *p*<0.001	0.30 (0.29–0.31), *p*<0.001	0.12 (0.12–0.13), *p*<0.001
	≥3 doses	0.97 (0.94–1.00), *p*=0.073	0.27 (0.25–0.29), *p*<0.001	0.22 (0.19–0.26), *p*<0.001

Figure S1: Conceptual model of confounding variables in the analysis of disability and COVID-19 risk.

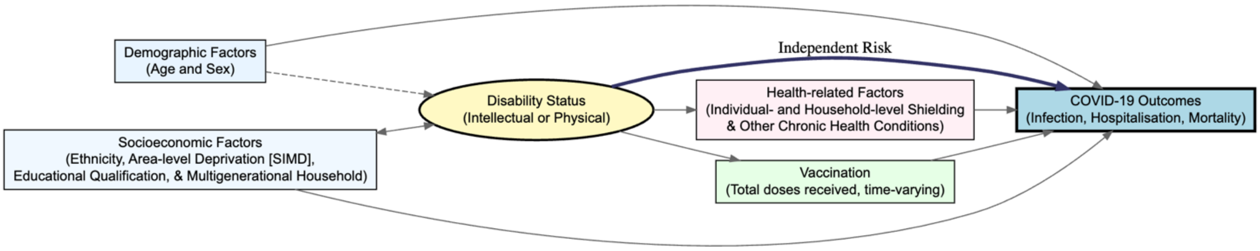

Figure S2. Crude incidence rates of COVID-19 outcomes by disability status

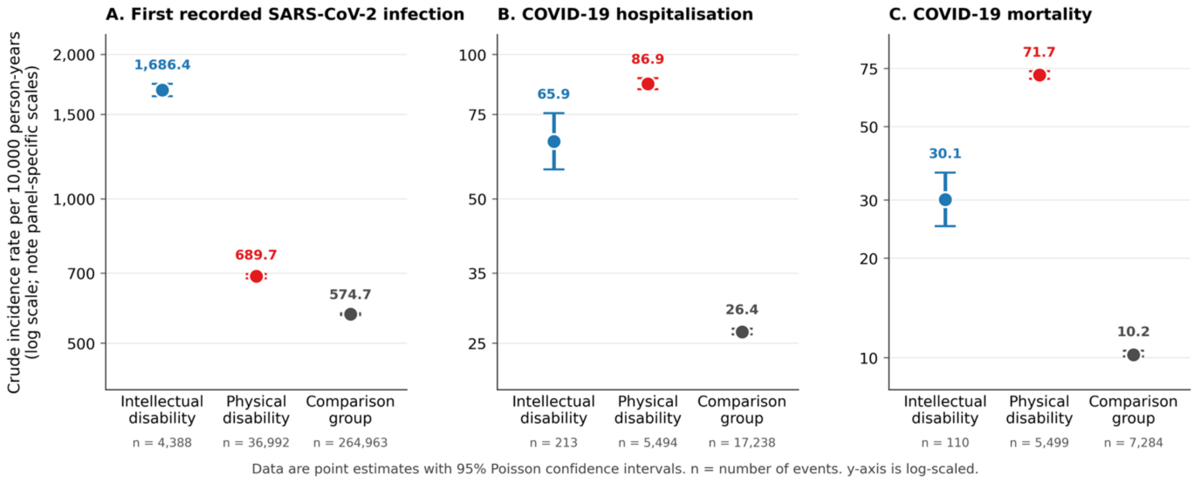

Crude incidence rates per 10,000 person-years for (A) first recorded SARS-CoV-2 infection, (B) COVID-19 hospitalisation, and (C) COVID-19 mortality, by disability status. Points are point estimates; error bars are 95% Poisson confidence intervals. Y-axis is log-scaled with panel-specific ranges. Numbers below each marker indicate event counts. Crude rates do not adjust for differences in age, sex, ethnicity, deprivation, or other characteristics across disability groups; for example, the physical disability group is markedly older than the intellectual disability group, which contributes to the higher crude rates of severe outcomes observed for physical disability. Adjusted hazard ratios are presented in Table 3 and Figure 2.

Table S5. Sensitivity analyses excluding intellectually disabled participants with upper secondary or higher qualifications

Outcome	Disability status	Main model<br>Adjusted HR (95% CI)	Excluding ID participants with upper secondary or higher qualifications<br>Adjusted HR (95% CI)
**First recorded SARS-CoV-2 (COVID) infection**	Comparison group	Reference	Reference
	Intellectual disability	2.65 (2.57–2.73), $p<0.001$	2.65 (2.57–2.73), $p<0.001$
	Physical disability	1.60 (1.58–1.62), $p<0.001$	1.60 (1.58–1.62), $p<0.001$
**COVID-19 hospitalisation**	Comparison group	Reference	Reference
	Intellectual disability	1.60 (1.40–1.83), $p<0.001$	1.63 (1.42–1.87), $p<0.001$
	Physical disability	1.16 (1.12–1.19), $p<0.001$	1.16 (1.12–1.20), $p<0.001$
**COVID-19 mortality**	Comparison group	Reference	Reference
	Intellectual disability	1.58 (1.30–1.91), $p<0.001$	1.60 (1.32–1.94), $p<0.001$
	Physical disability	1.23 (1.19–1.28), $p<0.001$	1.23 (1.19–1.28), $p<0.001$

Abbreviations: HR, hazard ratio; CI, confidence interval; ID, intellectual disability. Sensitivity analyses excluded participants classified as having intellectual disability who also reported upper secondary, post-secondary, or university-level qualifications. This excluded 907 participants, representing 5.2% of the intellectual disability group. Hazard ratios were estimated using Cox proportional hazards models adjusted for age, sex, ethnicity, area deprivation, highest qualification, multigenerational household status, individual shielding, household shielding, other chronic conditions, and COVID-19 vaccination status.
